# Between utopias and everyday practices: ecopreneurs and new urban economies in Bologna

**DOI:** 10.3389/fsoc.2026.1941048

**Published:** 2026-09-04

**Authors:** Marianna Musmeci, Laura Sartori

**Affiliations:** Department of Political and Social Sciences, University of Bologna, Bologna, Italy

**Keywords:** distinctive consumption, economic activities, everyday practices, real utopias, sustainable entrepreneurship

## Abstract

This article examines how New Urban Economies (NUEs) contribute to, and reveal the contradictions of, socio-ecological transition in Italian cities, using Bologna as a case study. It brings together three strands of research that are usually treated separately: studies of distinctive and environmentally oriented consumption practices; sustainable entrepreneurship research combining supply-side and demand-side explanations; and scholarship on temporality, prefiguration, and everyday utopias. Integrating these perspectives allows us to shift attention from macro-level sustainability indicators to the micro-relational and organizational practices through which socio-ecological change is interpreted, negotiated, and enacted. NUEs are defined as innovative market-based activities that systematically combine economic viability with environmental and social sustainability. Their originality lies not simply in offering green products or services, but in experimenting with alternative ways of organizing economic relations within the urban context. The empirical analysis draws on 23 semi-structured interviews with founders of small businesses in the food and clothing sectors. Three main findings emerge. First, becoming an ecopreneur often represents a biographical turning point through which individuals seek coherence between critical consumption practices, personal values, professional aspirations, and concerns about climate change and social justice. Second, doing ecopreneurship involves the continuous negotiation of sustainability in everyday organizational choices, particularly in supplier selection and relationships with employees and customers. Third, the transformative potential of ecopreneurship does not lie in eliminating contradictions or producing immediate systemic change, but in turning such contradictions into sites of practical experimentation. The article argues that NUEs can be understood as everyday utopias: situated micro-scale economic experiments that connect past experiences, present constraints, and imagined futures. Rather than attributing heroic agency to individual entrepreneurs, it shows how NUEs make alternative economic relations locally practicable while exposing the structural limits, uneven responsibilities, and institutional weaknesses of urban socio-ecological transition in contemporary Italian cities.

## 1 Introduction

As ecological awareness rises, the individualization of responsibility and the trend of green consumerism have made environmentalism a distinguishing factor for consumers.

This shift indicates a similar trend in sustainability entrepreneurship, often referred to as “ecopreneurship.” Within this context, the complex layers of pro-environmental attitudes play a crucial role in guiding entrepreneurial initiatives and distinctive consumption practices that promote sustainability. In addition to the well-known triple bottom line model (3BLM), which emphasizes the connection between people, planet, and profit (Muñoz and Cohen, 2017), this layered aspect also influences the narratives that entrepreneurs create. These narratives reveal profound ethical, systemic, and ideological beliefs. In the realm of economic activity, new narratives are emerging that act as catalysts for change in daily life, shaping the identities of ecopreneurs and guiding their actions. Narratives also support the concept of utopia, which proposes alternative models for how society could function based on the latent potentialities of the present (Levitas, 2013). These potentialities are primarily identified in the contradictions of capitalism (Wright, 2010) to which alternative models respond through transformative everyday practices (Cooper, 2014). By reimagining and redefining economic practices, sustainability entrepreneurs innovate to realize their visions for the future. Projects such as complementary currencies (Sartori, 2020), trade circuits (Zelizer, 2004), and local exchange trading systems (LETS) can therefore be interpreted as new economic practices that sustain “real utopias” (Schlange, 2009), fueled by a concrete tension between dreams and practices. Economic actors first conceive of these projects and articulate their practical potential, then experiment with “everyday utopias” through their everyday practices (Cooper, 2014). This is why ecopreneurs think in transformative—and sometimes even utopian—ways. How can we solve the consequences of climate change while also initiating profitable economic activities? How can we understand the changes in the world and reconsider social expectations and economic needs? What can we do today to help preserve the environment and make a living? In their daily lives, ecopreneurs blend the ordinary with the utopian.

This article adds to the line of research investigating novel forms of economic action by shifting the focus to the micro-level of everyday practices where distinctive biographical features, organizational traits of running sustainable business, and alternative visions of the future blend together. We identify these transformative practices as New Urban Economies (NUEs), characterized by innovative, sustainability-focused ideas in the trade and services sector.

We argue that NUEs can exemplify urban-driven sustainable economic actions and represent a form of everyday utopias, which are a micro-scale instantiation of “real utopias.” These practices are transformative not because they seek to resolve the contradictions of capitalism at a high general level, but because they transform these contradictions into the object of everyday experimentation. Indeed, we regard NUEs as “everyday utopias,” that is, “micro-instantiations of real utopias.” Therefore, we explore Bologna as a case study to problematize the social, economic, and institutional features conducive to such innovative forms of urban economic action.

The structure of the paper is as follows: in Section 2, we draw on three strands of literature (on social distinction, sustainability entrepreneurship, and temporalities) to capture the essence of NUEs as ecopreneurs to experiment with distinctive, sustainable, and transformative practices. In Section 3, we outline the case study and the research design. In Section 4, we utilize empirical material to show how becoming, doing, and making ecopreneurship relate to new forms of urban economies. Finally, we discuss our findings and present the conclusion.

## 2 The embeddedness of sustainability in the urban context

The city has always played a crucial role in the emergence, institutionalization, and transformation of capitalism. From “growth” (Logan and Molotch, 1987) to “innovation” machine (Florida et al., 2017), the city has served as an incubator and “nursery” (Duranton and Puga, 2001) for entrepreneurial activity in the industrial and service sectors. Urban areas continue to remain critical sites of production and consumption, as knowledge-intensive service activities tend to cluster in urban cores and are location-sensitive (Melo et al., 2009). Firms and small businesses are place-based and deeply embedded in specific localities, with their ownership, resources, and activities all rooted there. This connection gives them a sense of place and social mission (Shrivastava and Kennelly, 2013). While often depicted as abstract and disconnected, the concept of sustainability can be enriched by institutional and spatial perspectives. This aspect has been overlooked in entrepreneurship research (Vale et al., 2024). As sustainability challenges market fundamentalism (Block and Somers, 2014), the urban context provides an ideal environment for experimenting with innovative production and consumption methods that aim to advance sustainability.

To capture the embeddedness of sustainability in the urban context through ecopreneurs' practices, we draw on three established areas of literature. The first stream examines distinctive consumption practices; the second concerns sustainable entrepreneurship; and the third investigates the transformational potential of ecopreneurs' practices. This approach enables us to focus on the everyday practices of ecopreneurs through which they realize their vision, combining environmental sustainability and viable livelihoods. In Section 4, each of these three strands of research will be underpinned by empirical findings showing how ecopreneurs come to be (becoming), how they run their businesses sustainably (doing), and how they put their vision of a sustainable future into practice (making).

### 2.1 Distinctive practices of consumption

As a social group, consumers exhibit distinctive consumption styles shaped by their economic and cultural capital. Economic capital encompasses wealth, property, and financial assets, while cultural capital includes education, tastes, and intellectual orientation (Bourdieu, 1984). Individuals who engage in environmentally conscious consumption practices tend to possess higher cultural capital rather than economic capital (Carfagna et al., 2014). The upper middle class, characterized by high cultural capital, has deeply ingrained ecological values, making sustainability a core aspect of their lifestyles. This has led to the development of an “eco-habitus,” where sustainability influences practices across various life domains (Kennedy and Givens, 2019; Ginsburger, 2020). These individuals adopt and prioritize specific symbolic practices, such as eating a vegetarian diet, reducing air travel, and supporting green taxes, which can coexist with market-oriented economic activities.

As consumers are increasingly viewed as the hybrid figure of the citizen-consumer (Clarke, 2007), we link this concept to that of the entrepreneur. The identity of the citizen-consumer is often expressed through their market choices, which can highlight possible contradictions with the ideal of sustainability, which is often framed more as a corporate value than a characteristic of citizenship (Johnston, 2008). Sustainability entrepreneurs are critical consumers with a specific eco-habitus who seek to leverage the choices and freedoms offered by the neoliberal economic system in socially, economically, and environmentally transformative ways (Ginsburger, 2020).

### 2.2 Sustainable entrepreneurship research

Entrepreneurship is not solely dependent on individual traits; it is also heavily influenced by social structures, networks, and institutional contexts, which combine the supply and demand sides. Consequently, a variety of empirically driven analytical tools have demonstrated the different forms of entrepreneurship that have emerged in recent decades. These include alternative economic practices and community-based initiatives that are seen as alternatives to capitalism (Gibson-Graham, 2008; Sánchez-Hernández and Glückler, 2019), as well as purpose-driven urban entrepreneurship. The latter involves public-private collaborative initiatives aimed at enhancing citizens' urban well-being (Cohen and Muñoz, 2015). Other examples include eco-, social-, institutional-, and community-based entrepreneurship (Schaltegger and Wagner, 2011).

Recent research has expanded the understanding of entrepreneurship by integrating both supply-side and demand-side approaches. The former focuses on personality traits, cultural influences, social class, and ethnic group characteristics that encourage entrepreneurial ambitions and activities. Conversely, the latter explores the institutional and social context of economic activity, highlighting the opportunities available to entrepreneurs, driven by consumer demand, social networks, and environmental factors (Botelho et al., 2024). In the area of sustainability entrepreneurship, two aspects are particularly relevant to our objectives: the origin of entrepreneurial activity and its context. The first draws on supply-side research to identify several factors that contribute to sustainable entrepreneurship. For instance, much research has examined intentions and motivations related to green investment, as well as self-efficacy, a crucial factor in recognizing sustainable entrepreneurial opportunities (Halberstadt et al., 2024).

The second point draws on demand-side research to examine the contextual factors that influence the initiation of entrepreneurial activities. These factors include formal and informal institutional structures that shape the process of identifying opportunities and recognizing risks (Muñoz and Cohen, 2017), relationships with stakeholders (Schlange, 2009), policies and government incentives, market entry strategies, technologies (De Clercq and Voronov, 2011), cultural endeavors, and supportive social contexts (Muñoz and Dimov, 2015).

Entrepreneurship exhibits distinctive features regarding sustainability. While it is accurate to say that sustainable entrepreneurship is a form of social or environmental entrepreneurship, research also shows that it is a distinct field. This area examines the role of entrepreneurial action as a mechanism for sustaining nature and ecosystems while providing economic and non-economic gains for investors, entrepreneurs, and societies (Shepherd and Patzelt, 2011; p. 138).

Thus, connecting sustainable entrepreneurship and local contexts is relevant. Shrivastava and Kennelly (2013) emphasize the relevance of the territorial dimension for sustainability-oriented enterprises, which they refer to as Place-Based Enterprises (PBEs). These enterprises act as strategic actors within local systems for innovation and sustainability, combining market orientation with the wellbeing of their territory. This type of entrepreneurial activity, also known as the 3BL model, does not pursue social, economic, or environmental goals separately (the triple bottom line of sustainable activity). Instead, it integrates these goals within an urban organization that is “sustainable in its goals and in its method of generating wealth” (Young and Tilley, 2006; p. 88). Achieving this integration requires compelling narratives and, as we will argue in the next section, everyday practices that prefigure the desired change.

### 2.3 The temporal dimension of ecopreneurship: making sustainable futures through everyday practices

To study New Urban Economies (NUEs) as emerging forms of economic activity at the urban level, it is relevant to examine how economic actors organize their everyday practices, particularly in relation to their temporal nature. Temporality is a crucial dimension in this discussion, as highlighted by constructivist and phenomenological sociologies of time (Schütz and Luckmann, 1974; Adam, 1990; Zerubavel, 1985). However, studies on sustainability entrepreneurship often neglect the temporality of economic actions, even though the most common definition of sustainable development explicitly links the present and the future. The Brundtland Report states that sustainable development “meets the needs of the present without compromising the ability of future generations to meet their own needs” [World Commission on Environment and Development (WCED), 1987]. This oversight results in an incomplete understanding of the phenomenon (Bansal and DesJardine, 2014). This shortcoming stems from the field of economics' greater focus on macro-level dimensions rather than the micro-relational dynamics. As Hayes (2024) notes, scholars have largely focused on a macro-level analysis of how time shapes formal institutions (Polanyi, 2002; Miyazaki, 2003; Hirschman, 2021), labor markets (Adam, 2003), production models (Hassard, 2017) and consumption patterns (Shove, 2020), while neglecting micro-relational dynamics (Zelizer, 2012; Bandelj, 2020; Beckert, 2016).

Drawing on the relational perspective, some authors refocus attention on time and the agency of economic actors at a micro level. Hayes (2024) argues that time should not only be seen as a limitation but also as a strategic resource that social actors use to create, maintain, or change social bonds through economic activities. The author distinguishes between time as a structure, which refers to predictable cultural models for coordinating transactions and attributing meanings to relationships, and time as a dynamic process, in which actors strategically shape timing, duration, sequencing, pacing, and synchronization to signal intentions, manage expectations, and exercise temporal power. An example can be found at farmers' markets, where cyclical time (seasons and the rhythm of agricultural life) supports traditions and fosters lasting bonds. Sellers and customers coordinate their schedules around market days, during which the setup of stalls, sales, and community exchanges becomes intertwined with the ongoing narrative of community life.

This paper aims to refocus attention on the significance of the temporal dimension in sustainability studies. It employs the concept of “temporal work” (Kaplan and Orlikowski, 2013), which is defined as “any individual, collective, or organizational effort to influence, sustain, or redirect the temporal assumptions or patterns that shape strategic action” (Bansal et al., 2022; p. 7). The paper demonstrates how practices, discourses, and material objects that arise in everyday activities influence the temporal structures that coordinate both individual and collective actions. These contributions clearly demonstrate that economic actors do not merely endure temporal constraints but actively engage with and redefine these constraints to coordinate action, infuse their interactions with meaning, and negotiate power relations. To understand how everyday practices help economic actors strategically link the past, present, and future, three important elements need to be considered.

The first is the concept of “imagined futures” (Beckert, 2016). When faced with high levels of uncertainty, actions cannot be based on probabilistic calculations, but rather on so-called “fictional expectations”: representations of the future that are neither true nor false, but which are credible and capable of mobilizing resources and influencing decisions. Similarly, Rindova and Martins (2022) use the concept of “futurescapes,” which highlights how firms can expand the boundaries of what can be imagined and pursued. Futurescapes are discursive constructions that alter perceptions of what is possible and desirable, thereby broadening the range of strategic options available to economic actors such as entrepreneurs. The ability to invent the future is linked to the capacity to aspire to and imagine different possibilities (Appadurai, 2004), but this ability is unequally distributed among individuals. A complex combination of economic, social, and cognitive resources affects an individual's ability to express and cultivate their aspirations for the future, as well as to see those aspirations recognized. In this context, certain social groups (such as those with more cultural than economic capital) may be more inclined to imagine new forms of economy within urban settings that could transform the existing economic structure.

The second element draws on studies of prefiguration (Monticelli, 2018; Zanoni et al., 2017), which illustrate how representations of the future are formed in the present. Prefigurative organizations do not merely imagine an alternative future; they also construct the changes they aspire to in the present through practices that reject dominant organizational forms and experiment with different modes of production, exchange, and governance.

The third element draws on the concept of everyday utopias (Cooper, 2014), which helps us identify spaces where this prefigurative mechanism takes shape. By observing the ordinary fabric of these spaces—composed of repeated, seemingly mundane gestures, interactions, and concrete choices—we can better understand how everyday utopias are realized through situated practices.

These utopias are not merely dreams or abstract projects; they are tangible practices that foster environments (both physical and symbolic) in which the future is actively enacted, rather than awaited or described. Cooperatives, exchange communities, and care networks are a few examples of spaces where everyday utopias unfold.

These are spaces where everyday activities are carried out in alternative ways that incorporate egalitarian, democratic, or emancipatory principles. Unlike past visions of utopias, the everyday utopia is situated in the present. It is rooted in the interstices of daily life and the capacity of individuals and groups to redefine and imagine new ordinary practices. In other words, it is a type of utopia that recognizes everyday life as the frontier of what is possible (Wright, 2010). Utopias are rooted in the potential of individuals and take the form of alternative experiences that can bring about change in the structures of power and domination. The tension between dreams and practices fuels real utopias; what is pragmatically possible is shaped by our visions. This set of situated practices is driven by ecopreneurs' ability to envisage the future and underpinned by the transformative potential of everyday practices—that is, choices made, options tried out, and actions rooted in the present. The utopian impulse emerges in everyday life (Jameson, 2005), linking the present and the future. It is through practices that challenge the ordinary—the element we take for granted—that our ecopreneurs can envisage new forms of urban economy (NUEs). By exploring the intersection of these three areas of literature, we can gain a deeper understanding of the motivations for becoming ecopreneurs and the everyday practices that, by fostering innovation in economic activities, enable us to experience the transformative potential of NUEs.

## 3 Case study and methods

### 3.1 Bologna as a laboratory for NUEs

This article analyses the case of Bologna, a medium-sized city in northern Italy with a population of around 400,000 (Istat, 2026). A symbol of the so-called “Third Italy” (Bagnasco, 1977), Bologna is characterized by a development model based on clusters of small and medium-sized enterprises, organized into networks of subcontracting and cooperation, specializing in high-quality production and capable of responding flexibly to market demands. The city is situated in one of the country's wealthiest regions. It is the hub of the Emilian Model (Battilani and Zamagni, 2012): a development model in which the local administration has historically combined welfare policies with support for industrialization and SMEs within a broader political and institutional framework of communist origin. However, since the 1990s, the Emilian model has been progressively eroded under pressure from various interrelated factors: the crisis of the communist ideology that had served as a social glue; the growing shift toward market-driven approaches; the tendency of local authorities to adopt a managerial approach inspired by corporate efficiency; and the more recent processes of the city's “touristification,” which have accelerated urban commodification and housing inequalities (Giovanardi and Silvagni, 2021).

These changes are transforming Bologna into a “hybrid laboratory,” where market logic and the cooperative tradition coexist through constant compromise. The role of the local administration and the city's strong civic tradition mitigate the most disruptive effects of neoliberalism (Giovanardi and Silvagni, 2021). This capacity for mediation is also reflected in the sustainability policies implemented through comprehensive strategic planning, international commitments, and participatory experiments (Fontana et al., 2025). The 2018 Metropolitan Strategic Plan 2.0 explicitly endorses the United Nations' Agenda 2030, identifying sustainability as a pillar of the Bologna metropolitan area's development alongside inclusivity and attractiveness. Sustainability is broken down into three interconnected dimensions—environmental, economic, and social—and translated into targeted sectoral policies ranging from mobility to urban regeneration. The distinctive interplay between the economy, institutions, and civil society that characterizes the Bologna ecosystem therefore provides a prime vantage point for understanding how the NUE are developing within a productive fabric already geared toward flexibility and deep local roots.

### 3.2 Research design and methodological approach

This article presents the results of a qualitative analysis of the Bologna case study, based on semi-structured interviews with the founders of New Urban Economies (NUEs). The aim is to understand the organizational aspects of NUEs. NUEs are defined as innovative economic activities that operate in the market and systematically integrate environmental and social sustainability objectives. Our analysis focused on two key sectors: clothing and food. Businesses were selected through a mapping exercise of local enterprises that met the NUEs' definition. This involved combining a local keyword search (e.g., “sustainable business,” “solidarity economy,” “short supply chain,” “ethical fashion,” “organic farming”) with existing maps of the solidarity and sustainable economy. Examples of these maps include Greenpeace's Green City Map (https://www.greenpeace.org/italy/storia/13764/e-online-la-nostra-eco-mappa-per-vivere-la-citta-in-modo-sostenibile/), Italia che Cambia (https://www.italiachecambia.org/mappa) and the Italian Solidarity Economy Network (https://rete-ries.it/). The businesses identified in this way were then cataloged and subjected to field verification to confirm their existence and assess their relevance to the research objectives. This phase of dataset validation was followed by its conversion into an interactive map using the Google My Maps online tool, which enabled the selected businesses to be geolocated and categorized.

The entrepreneurs interviewed were selected through purposive sampling to reflect the diversity of new enterprises in Bologna. A total of 23 semi-structured interviews were conducted with small business owners operating in the clothing (9) and food (14) sectors in Bologna (see Table 1). The different sample sizes reflect a deliberate strategy: clothing, more homogeneous in its dynamics, was sufficiently captured with fewer interviews; food, by contrast, required a broader exploration to account for its internal diversity.

**Table 1:** Participants by age, gender, sector, year of foundation, and interview date.

Interview ID	Age	Gender	Education degree	Sector	Year of foundation	Interview date
P_1	34	M	Bachelor	Food	2013	13/12/2025
P_2	54	F	High school	Food	2020	14/01/2025
P_3	63	F	Master	Food	2016	21/03/2025
P_4	49	M	Master	Food	2019	11/01/2025
P_5	36	F	Master	Clothing	2021	05/02/2025
P_6	34	F	Master	Clothing	2014	24/01/2025
P_7	41	F	Bachelor	Clothing	2018	12/02/2025
P_8	65	F	High school	Food	2003	17/03/2025
P_9	39	M	Bachelor	Clothing	2014	12/02/2025
P_10	46	F	High school	Clothing	2012	16/01/2025
P_11	30	M	High school	Food	2022	17/12/2024
P_12	28	F	Bachelor	Clothing	2022	28/01/2025
P_13	44	F	High school	Food	2021	30/01/2025
P_14	56	F	Master	Food	2013	21/01/2025
P_15	42	M	Master	Food	2015	22/01/2025
P_16	40	F	Bachelor	Clothing	2009	25/02/2025
P_17	45	M	PhD	Food	2023	06/12/2024
P_18	57	F	Master	Clothing	2010	21/02/2025
P_19	29	M	Bachelor	Food	2021	30/01/2025
P_20	44	F	Master	Clothing	2023	20/02/2025
P_21	35	M	Master	Food	2021	28/02/2025
P_22	43	M	Bachelor	Food	2021	04/03/2025
P_23	37	M	High school	Food	2019	27/02/2025

To gain insight into the interviewees' entrepreneurial experiences, the interview guide was structured around four main analytical dimensions. (1) Biographical: aimed at reconstructing life stories in relation to education and work, paying particular attention to work values, perceived job satisfaction, and the relationship between private and professional life. (2) Organizational: aimed at examining the genesis of the business idea, the role of the local area, and the personal networks activated during the start-up phase. It also examines the methods of resource acquisition, the composition of the business, and any changes to the initial value orientation. (3) Market: aimed at investigating the enterprise's awareness of its target customer base and its supply network. (4) Institutional: aimed at understanding the role of local institutions and public policies in supporting or hindering these entrepreneurial activities. All interviews were rigorously transcribed and anonymized. A thematic analysis was then carried out using NVivo 14, the software available to the team. Through a recursive process of coding and interpretation, cross-cutting patterns of meaning were identified across participants' narratives. A critical lens was adopted, focusing on how participants discursively constructed their experiences.

## 4 Bologna as a social, economic, and institutional site for ecopreneurship

The key distinguishing factor between ecopreneurs and traditional entrepreneurs is their commitment to integrating economic, environmental, and social objectives. While ecopreneurs share a hybrid identity, they are not a homogeneous group; several distinct types have been identified (Halberstadt et al., 2024). Our contribution enhances the understanding of the various individual, organizational, and institutional factors that drive ecopreneurship. Through our interviews, we provide valuable insights into three dimensions: personal biographies (4.1), organizational choices (4.2), and the transformative power of everyday utopias (4.3). Embarking on a life journey that confronts you with the limitations and contradictions between economic, social, and environmental objectives is an important step toward becoming an ecopreneur. These very contradictions motivate them to redefine what it means to do business sustainably, envisioning a different future that can be realized through their everyday economic activities.

### 4.1 Being an ecopreneur: between self-realization, social justice, and the institutional context

*I haven't bought fast fashion for years [..] it's madness when you think about climate change as well… I mean, mountains and mountains of clothes… to think they don't just disappear. It's not as if they vanish into thin air. People, even though we know all this now from films, documentaries and articles… well, even at Christmas, there were queues outside certain fast-fashion shops, like Zara. For me, though, my values really are that you shouldn't buy two thousand items, but just a few that you know you can mix and match throughout the season. [P_10]*

*Then what I like [about this job] is also pursuing a choice that's in some ways political—even though I'm not someone who wants to get involved in politics—but choosing a different diet, or at least a different way of consuming food, a different kind of purchase and a different way of shopping too; in my view, that's also putting a political ideology into practice. [P_13]*

The eco-entrepreneurs interviewed had already adopted critical consumption practices before setting up their businesses. As citizens and consumers, they base their purchasing decisions on criteria that extend well beyond price and utility. These criteria include perceived quality, the product's origin, production conditions, supply chain transparency and social and environmental impact (Micheletti, 2003; Sassatelli, 2006; Forno and Graziano, 2014). Drawing on their experience with daily consumption practices, these eco-entrepreneurs strive to align their business activities with environmental and social sustainability criteria while ensuring they meet operational needs and remain economically viable.

The first element common to the interviewees' narratives is therefore an “eco-habitus,” understood as a set of ecological dispositions distinctive of consumers with high cultural capital (Carfagna et al., 2014). This eco-habitus serves as the foundation for their motivation to engage in entrepreneurial activities. However, the decision to launch a sustainable business cannot be fully understood if eco-entrepreneurs are viewed simply as entrepreneurs with a keen awareness of environmental and social issues, or as activists who use the market for a specific purpose (York et al., 2016). Our interpretive coding of the interviews shows that none of our interviewees describes themselves as activists running a business, nor do they identify with the values that guide traditional entrepreneurs. Instead, they tend to present themselves as highly qualified professionals who combine economic considerations with personal passions, professional aspirations, and values centered on sustainability and social justice.

*I then completed a three-year economics degree, writing my dissertation on the sustainability reports of several large companies. This allowed me to combine my passion for fashion with the environmental and social sustainability that I had studied and enjoyed so much during my undergraduate degree. I then specialized further by taking a course in green fashion here in Bologna. I made my first garments in the summer of 2020. Little by little, I evolved, trying to strike a balance between environmental sustainability in terms of materials and economic sustainability so that my project could move forward and be financially self-sustaining. [P_12]*

For most of our interviewees, becoming an ecopreneur is primarily linked to the perception of an increasing disparity between personal values and lifestyles and the logic of the salaried labor market, as well as the production and organizational practices of large corporations. While this does not necessarily lead to an immediate entrepreneurial venture, perceiving this discrepancy in terms of identity makes it challenging to continue in career paths considered inconsistent with one's values and daily practices. Therefore, it is not surprising that most respondents attribute their decision to become an ecopreneur to the need for greater consistency between their values, daily practices, and professional aspirations.

*Before setting up the company, I worked in the United States—I lived there for a good 13 years—where I was always involved in fashion, but in a more conventional way. So it was a very large company, and I was in charge of buying [...] but after so many years in that sort of job, even with a very conventional approach to fashion… meaning a lack of focus on certain aspects… it eventually leads you to mature, perhaps, and decide to strike out on your own. [P_7]*

*I went round a bit, doing some interviews at some very important and well-known large companies we have here in Bologna. However, I was completely opposed to joining a company, even though you certainly learn a lot from a technical perspective. [...] You see the pattern on paper, then the finished garment arrives, you check for faults—can you do that? I do not know, who knows, because you did not actually sew that garment yourself. And then, yes, the fact that they all had their production outsourced, the way they treat their employees… I mean, I have met people who have worked for or are working at these companies, and the approach isn't always positive or respectful of working hours, needs, and requirements. [P_12]*

For some respondents, the decision to become an ecopreneur was influenced by life-changing events—disruptions at an individual level (job loss, the end of a romantic relationship, the loss of a family member, etc.) or at a collective level (economic crisis, war, pandemic, etc.)—which led them to redefine their life path significantly. As we shall see in the following excerpts, the search for greater authenticity is linked to the moral responsibility that leads the individual to take personal responsibility for major global challenges—from the climate crisis to social justice.

*At a certain point during the courses I was taking at the workshop, I was sacked by the company I'd worked for eight and a half years. In the meantime, I'd completely changed my diet; I'd become almost entirely vegan [...] [The opportunity to take over the workshop] also came at a time when I really wanted to change my lifestyle a bit – and perhaps, to some extent, this is something I think about from time to time: making amends for what I'd done before. It's a bit like saying: I'm trying to do something a bit more virtuous now. Whereas before I was creating rubbish, making junk, now I'm trying to save resources. [P_13]*

*After that I finished school and started working for a fashion design studio for four and a half years [...] when 9/11 happened, that marked a bit of a turning point in my life, because just as it was happening, I was at a fabric fair in Milan, at Moda In, and right then and there, seeing what happened… [...] I said to myself: I'm doing the most pointless job in the world. It was like a wake-up call, and from then on, I decided to change careers completely, even though I loved it so much; I wanted to do something useful for others. [P_10]*

In terms of identity, setting up a sustainable business is a significant turning point that can bring coherence and continuity to people's life stories. Additionally, it serves as a structured response to the labor market crisis and prevailing economic logic. Sustainable entrepreneurship is a means of realizing professional aspirations without compromising values and ideals, even if it is not the ultimate goal (Azzolina and Mazzenzana, 2026).

The desire for professional fulfillment is intertwined with past experiences and the relevant local and institutional context in various ways. This context often struggles to recognize and support the transformative potential inherent in this approach to business. The analysis shows that the focus on sustainability takes on different nuances, reflecting the complexity of individual life paths. While all interviewees appear to be aware of a collective dimension to their entrepreneurial project, different understandings of business sustainability emerge depending on their cultural background and the experiences they have gained through associations, trade unions, or politics. Notably, those with participatory experience tend to link environmental sustainability to social justice.

*Clearly, I have always been driven by ideas of justice and ethical working practices. This seemed to me to apply to the world of commerce. You might think that people turn up their noses at commerce because it involves money, but that's not really the case, because we all have to earn a living. However, I wanted to offer a product based on different, more ethical ideas. [P_14]*

Individuals with technical or business backgrounds often take a practical approach to sustainability, focusing mainly on optimization and efficiency. In this context, sustainability is not associated with broad claims about collective interests. Instead, it reflects an entrepreneur's aim to clearly showcase their ability to minimize the economic impact on both the environment and society.

*From the outset, we have placed a strong focus on sustainability. In collaboration with the Metropolitan City, we even won the Barresi Award for sustainability. We have also carried out various surveys and assessments with the Region to measure the company's impact. Sometimes people think of sustainability simply in terms of recycling, but it's a whole world in itself. So let's consider it from a governance perspective—that's the term I use. Bear in mind that although we are an SME, we have a protocol in accordance with Law 2231, which involves auditing all our processes. We established this right from the start by adopting that approach. I am also certified as an SG Advisor, and all of these aspects stem from the training I received in my previous role. [P_22]*

The drive toward entrepreneurship does not occur in isolation but rather draws its strength and legitimacy from the local and institutional context. The analysis shows that most respondents perceive Bologna as “fertile ground” for this type of entrepreneurship. It is often described as a “hub,” providing a supportive environment for establishing and developing one's own business venture.

*[...] we've opened here—which, in any case, is a city that, quite apart from its central location, is becoming something of a hub for certain things. So it's a bit of a challenge, because it's still a city that, in some respects, remains a bit provincial; yet it's certainly a city that now has a wide catchment area, not least thanks to the tourism it's seen in recent years—even post-Covid, I'd say. [P_7]*

*If I'd done something like this in Modena, I honestly don't know if I'd even still be in business; Bologna is certainly very fertile ground for a project like this. It's probably fertile ground for a café too, but a project like this is right up its street.[...] This place is a ticking time bomb, it's a feeding ground; so just put “vegan” on the sign and we'll be there. [P_23]*

Although the respondents were able to identify and capitalize on market opportunities in the Bologna area, local policies that encourage and support this type of business present several challenges. While the interviewees recognize the potential of local and regional authorities to foster the launch and growth of sustainable businesses, many feel that the support provided is overly selective, fragmented, and difficult to access. Current support mechanisms for sustainable entrepreneurship are limited to specific types of business, such as those in the craft sector, while those in the catering sector are excluded. Furthermore, only a minority of our respondents have benefited from these measures. Applying for funding requires time, financial capital, and technical and administrative skills, which small businesses often lack, especially during their start-up phase.

*The state doesn't help women—because that's a lie—nor does it help those who want to invest in a sustainable economy. [P_2]*

*I think ordinary people have supported us more than the institutions… now, fortunately, we've won Incredibol. I do think, though, that Bologna isn't a bad city. Let's just say I was expecting much more—much more funding for young women—because I started under 30, I registered for VAT under 30, and nobody gave me a single euro. Even though I was looking for calls for proposals, with European calls you need a consultant who's paid a fortune—you probably know that better than I do—and even now we had a European call to hand that we wanted to apply for, but you absolutely had to have a consultant to take part, and we didn't have the budget for that at the time because, after all, you also need the cash to pay them. [P_5]*

### 4.2 Doing ecopreneurship in everyday life

Ecopreneurs operate within an economic system where the pursuit of profit often conflicts with environmental protection and human rights. They find ways to navigate these conflicts and address the challenges of sustainable entrepreneurship, providing a new perspective on the economy and the role of entrepreneurs in society. Our analysis identifies three key areas where ecopreneurs manage these competing priorities: supplier selection, employee relations, and customer relations. By examining their everyday practices, we can see how ecopreneurs strive to achieve profitability while still upholding environmental and social objectives.

#### 4.2.1 Supplier selection

According to the interviewees, conflicts arising from pursuing different approaches mainly center on supplier selection, a critical decision that shapes the company's sustainability principles. Choosing suppliers is not just a procurement task or a neutral action; instead, it requires a careful evaluation that goes well beyond mere cost or efficiency considerations. This process involves a variety of factors that collectively define sustainability. These factors include production methods (organic or otherwise), the scale and intensity of production, geographical distance and mode of transport, packaging, transparency of information, formal certifications, and the personal relationship built up over time. Supplier selection cannot be fully understood as the mechanical application of unambiguous, standardized sustainability assessment criteria. Ecopreneurs draw on their personal and business backgrounds, the sectors they operate in, and the resources at their disposal to create a map that reflects their priorities and tolerances. They choose and prioritize the elements that contribute to product sustainability based on specific, relevant systems. As a result, supplier selection becomes a situated and relational process that emerges from a continuous negotiation between ethical principles and economic constraints.

*The suppliers of the products I sell in the shop are all companies, both Italian and international but generally from Northern Europe, that share the same philosophy. The poncho company, for example, is Danish. What do they do? They make a poncho from 20 recycled plastic bottles. The product is manufactured in Asia. It's a certified supply chain—you can scan the QR code to read the full story. In short, as I was saying, they must share the same philosophy, but they don't necessarily have to be based just two kilometers from my business. [P_7]*

For this interviewee, the primary selection criterion is the use of recycled raw materials. Her commitment to sustainability means she relies on overseas suppliers, prioritizing the guarantees offered by formal certifications over geographical proximity within the supply chain. In other cases, it is the relationships of trust established with suppliers and customers that take precedence over formal certifications.

*To be an organic shop that advertises “organic” everywhere, you need a certification that costs money to obtain. I decided not to incur this extra cost, so there was no mention that the products were organic. However, they were organic, so I told people they simply had to trust me rather than rely on a certificate. [P_14]*

Some interviewees believe that the authenticity of their entrepreneurial approach lies in personally verifying the quality of their products, regardless of whether they hold formal sustainability certifications. This practice anchors sustainability principles in the eco-entrepreneur's personal responsibility, thereby shifting away the focus of credibility and trust from technical compliance toward direct experience.

*The first thing I did was visit SANA and various trade fairs, and then I did some online research. I specifically looked for companies that don't use plastic in their packaging. I also make sure that they are as sustainable as possible. For example, I only choose detergents if they are biodegradable and all the products must be organic. I taste all of them to make sure they are good. [P_2]*

When researching and selecting suppliers, eco-entrepreneurs do not lose sight of market dynamics. Rather than getting lost in abstract idealism, they navigate the challenges of competitiveness, constantly balancing economic needs against ethical principles. The long-term viability of the business venture is inextricably linked to adopting sales strategies that ensure product competitiveness; otherwise, they risk being excluded from the market altogether.

*I usually try to stock unique products, so exclusivity is important to me. As soon as a niche product I have chosen becomes commonplace, I remove it from the shelves and look for another. [P_8]*

When choosing suppliers, ecopreneurs prioritize the working conditions of employees in the companies from which they source their goods. Our interviewees identified this as a crucial factor for social sustainability. Neglecting to consider this aspect can jeopardize the authenticity, consistency, and credibility of a business venture.

*Back then, I travelled all over Italy to find various suppliers and, above all, to see for myself how the companies operated. I really did see everything. For me, one of the most important things has always been knowing that the places where I buy clothes are places where people work and aren't exploited, because otherwise it isn't consistent with the message you're conveying about your product by embracing the whole ethos of vintage. [P_10]*

Sustainability moves from being an abstract concept to a practical challenge when it becomes difficult to pursue its three dimensions—economic, environmental, and social—simultaneously. For instance, sourcing from short supply chains while keeping prices affordable can create conflicts. The practical limitations of achieving “pure” or “radical” sustainable entrepreneurship often led to compromises. However, this should not be viewed as a betrayal of sustainable principles; rather, it is a necessary approach to ensure business continuity and maintain product accessibility.

*Products are organic and local, but many are sourced from organic distributors. I knew how they operate, but it's difficult to always maintain a good balance between quality and price. I'd made up my mind, but over the years I've learned that for certain products I should turn to reliable distributors, which meant lower costs for some items. For other, more specific and perhaps slightly more niche products, however, I should source locally. If I had sourced everything locally, I would have had to raise the prices so much that it wouldn't have been commercially viable. So, I had to strike a balance, but always with a clear conscience—everything was fully disclosed. [P_14]*

Another interviewee selected suppliers based on direct knowledge, not only to demonstrate the company's consistency with its ethical principles but also to facilitate intersubjective recognition (Honneth, 2019), thereby going beyond the instrumental function of supply networks and reintegrating social ties into the heart of economic exchanges.

*[The suppliers] I find them in the areas where I grew up. I've been selling at the Campi Aperti market for at least eight years, and I know everyone there. There is a person behind every product; I know how they work. So when I run out of chard, I get it from a friend or a nearby farm. The same goes for the other things I sell. Take drinks, for example: for me, behind every bottle there's a person and a strong bond. [P_23]*

In this context, entrepreneurial activity holds symbolic value that extends beyond the mere pursuit of profit. Sustainable entrepreneurship creates an alternative space to practice care, reciprocity, and responsibility. For the interviewee, entrepreneurship is deeply rooted in personal relationships. The supply chain is not just a series of anonymous, interchangeable suppliers; it is based on a clear understanding of production methods and working conditions. These relationships are built on trust and have been strengthened over time through shared experiences. While the market often leans toward impersonal and temporary exchanges, it is still possible to create economic relationships that are founded on belonging, reciprocity, and trust. This approach allows for resistance to the depersonalization typical of globalized supply chains. Therefore, there is potential to develop different economic practices that are both sustainable and personalized.

#### 4.2.2 Relationships with employees

Ecopreneurs address the challenges and contradictions of sustainable entrepreneurship in supplier selection by developing economic practices rooted in recognition and trust. The transformative potential of the New Urban Economy (NUE) is most evident in how they organize their employees' work. In their relationships with employees, ecopreneurs frame sustainability in moral terms. Decisions regarding wages, job security, and workload distribution highlight the tension between profit-driven logic and the enterprise's social sustainability. Ecopreneurs tackle this conflict through work organization models that move beyond the rigid hierarchies, precariousness, and subordination typical of contemporary capitalism. A key finding of the analysis is the prevalence of precarious employment and poor working conditions. An interviewee from the catering sector illustrates this point:

“*It may sound trivial, but offering permanent contracts or fixed shifts in the catering industry is a significant innovation, as many workers are often employed off the books. Some are paid for a single job, while others have to take on additional work.” The working environment can resemble that of Michelin-starred restaurants rather than typical street food establishments, which is our sector. For example, our delivery riders can sit indoors when it rains, and we provide them with drinks to foster a relationship. Over time, they have all become friends because we take the time to get to know them. [P_22]*

A common theme in discussions among ecopreneurs regarding their employees is the desire to provide decent working conditions. One interviewee (P_5) emphasized that offering a decent job involves attention to working hours, working conditions, and contractual stability. Another interviewee (P_22) focused on employee wellbeing, highlighting the importance of creating an environment that promotes a good work-life balance while also recognizing and valuing gender differences. This approach fosters a relationship with employees based on mutual recognition, which can lead to new forms of solidarity and care, moving the relationship beyond a purely instrumental focus on efficiency.

*One of our most recent recruits has an eight-month-old baby, so her time and working hours needs are very important to us. We prioritize the health of our employees and ensure that work does not dominate their lives—essentially, we aim to minimize work-related stress. Creating a pleasant working environment where our employees feel “safe” at work is crucial for us. We believe that not experiencing stress or competition with others is fundamental to our organization. Regarding our all-female team, I'm not exactly sure how to define this value, but it has recently become one of our core principles, and I view it as a very positive development. [P_5]*

The interviewee emphasizes the importance of relational and gender dimensions in work organization, framing them through the lens of care and moving away from a competitive mindset. She contrasts this with a mindset of mutual support, which is more aligned with organizational practices grounded in solidarity rather than rigid hierarchical structures.

There is a noticeable change in how businesses are structured, particularly in their decision-making processes. A restaurant owner's experience exemplifies this shift: he not only chooses not to exploit his position as owner but also explores innovative ways to redefine the relationship between owners and employees.

*I often find myself being portrayed—and even somewhat idolized—as a capitalist, which has absolutely nothing to do with my actions or experiences. Many people describe my workplace as unusual, but that's primarily because I never yell at anyone. I don't impose myself on others; I give people space. When someone has a different idea, I'm open to it. Sometimes, I'm even the one who gets told off. This approach is quite rare in many workplaces, where people exploit their positions to intimidate others. [P_23]*

In this context, we are not merely discussing a business owner who is open to dialogue and criticism. Instead, we see a concrete effort to make hierarchies less rigid, more flexible, and more negotiable. This is achieved by establishing “unconventional” workplaces where individuals are even willing to accept constructive criticism. Here, the limitations and contradictions of sustainable entrepreneurship are addressed by redefining the employer–employee relationship. This relationship is no longer based on subordination; rather, it is founded on mutual recognition and reciprocity.

#### 4.2.3 Relationships with customers

Ecopreneurs' desire to remain in the market without compromising their ethical principles creates tension in their relationships with customers, particularly when setting prices. Pricing is a crucial factor for a business's competitiveness and the consistency and authenticity of its venture.

This tension is evident in how ecopreneurs justify their higher prices to customers, unlike businesses that focus solely on profit. When customers complain or object, interviewees often change the subject to stress a different understanding of the product's value. This perspective emphasizes quality over cost and profit margins, highlighting the importance of raw materials and, more broadly, environmental and social sustainability.

*When customers come in and buy a kilo of washing powder, for example... Let me give you the example of the* €*15 washing powder. People tell me, “Yes, it's expensive.” It's true—it's expensive—but I pay* €*10 plus VAT for that washing powder, so ultimately it's expensive not because I want to make a huge profit. It's expensive because the raw material is expensive. [P_2]*

*[...] Sometimes, it's true, prices are higher, but that's due to certain products and also the cost of production. In my view, everyone must be able to afford it, and those who work must be paid fairly. So it's a difficult balance to strike. [P_1]*

By redefining the framework in which prices are set, eco-entrepreneurs overturn the logic of competition by shifting the focus from price to product quality. They also redirect customers' attention toward critical consumption, raising awareness of environmental and social sustainability.

*I'd never take part in sit-in protests on the motorway. I realize there's a need for that sometimes, too. But my approach is more subtle. It's about showing people that it's possible to eat well without meat, especially given the unsustainable practices of modern livestock farming. [P_3]*

*That was my aim too: if you want to eat in an environmentally responsible way and with a certain level of awareness, you can do so even if you don't have much money, because there's this idea that “organic” means “NaturaSì”—the supermarket for the rich and so on—which is partly true, of course, but I think I've done a great job of making it clear that everyone can afford to eat well without spending a fortune, simply by being mindful of what you buy. And for me… in fact, I've held several meetings on this very topic. [P_14]*

Notably, another interview shifts customers' focus from immediate convenience to responsibility toward the environment and future generations. This approach places economic exchange within a broader temporal and value-based framework.

*The prices are still high compared to those in the big supermarkets, and I always tell them, “Yes, that's true, but you also have to look a bit further ahead. I mean, you have children—I didn't even have the slightest idea I might have any myself before—and I always used to say: ‘I'm doing this for your children, not for mine, because I don't have any.' Perhaps you should start thinking about it too, about starting to shop in a certain way.” [P_13]*

### 4.3 Making ecopreneurship real: the transformative power of everyday practices

Practicing ecopreneurship requires making a variety of organizational decisions, such as choosing suppliers or setting prices that are not always aligned with sustainable entrepreneurship. Therefore, it is not surprising that many interviewees explicitly state that they “are not sustainable,” thereby highlighting the impossibility of controlling the environmental and social impact of the entire production chain and acknowledging inevitable trade-offs.

*We don't use any polluting products on our land, but then again, we do... because that's a point of view in itself, you see? Someone might say, “Yes, you don't use anything, but you use the tractor more, and that still uses fuel.” However, I don't use weedkillers, so I don't pollute my land, and I do everything mechanically. It's a long story, but that's our approach. In short, we try not to pollute. [P_1]*

*I do lots of things that move in this direction, but they're certainly not sustainable. The whole business isn't sustainable, in that we have three vans and everyone drives to work in Soliera. [...] How can you call this sustainable? We try lots of things, we provide a tray so we can wash it. Even here, I use only washable products that I couldn't otherwise use. I fight all my little battles, but we're up against a giant. So, in my view, we're not sustainable. [P_23]*

The interviewee continues his account, clarifying:

*Although we promote sustainability a great deal because we're still fighting on various fronts in this area. So, someone who isn't entirely inexperienced—or at least someone who believes in it—will certainly find a haven here rather than anywhere else. “I think that, of all the projects out there, especially the various vegan initiatives in the city, ours has a very strong ethical and social dimension.” [P_23]*

The difference between criticizing capitalism and recognizing the limits of one's own actions does not paralyze ecopreneurs. On the contrary, this awareness drives them to act, seeing the market as both a cause of and a possible solution to ongoing environmental and social crises. One interviewee describes her work as a “mission.”

*I feel I have a mission because promoting a plant-based diet involves many important aspects of humanity, such as our health and well-being, respect for animals, and the consequences for the planet. So, it is my mission. [...] I'm absolutely convinced that what we do is of great value. Whether or not we're recognized for it doesn't really matter to me. [P_8]*

Interviewees tend to describe their business activities as fertile ground in which to “sow” the changes they hope to see in the future. One such example is an interviewee who owns a clothing shop and organizes creative upcycling workshops.

*Working with recycled materials enables me to pursue the secondary aim of this project: engaging the local community. My goal is to encourage people to avoid throwing things in the bin and to make them reflect on which materials can be reused and which can be disposed of in a way that creates something that goes beyond my business itself and leaves a seed—the idea that perhaps we should pay more attention to what we throw away and how we use our resources. [P_20]*

In the interviewees' accounts, business is not viewed as a site of anonymous exchange, but as a relational space—that is, a place where economic transactions become an opportunity for encounters, meaningful interactions, and recognition. For example, one interviewee describes her shop as a “piazza” comprising encounters and forms of social interaction that prioritize the social and relational aspects of economic exchanges. This way of understanding and practicing economics, therefore, seeks to rebuild the fragmented social fabric by resisting the atomization imposed by neoliberalism.

*It's a completely different kind of shopping experience from the supermarket: there's a real need for interaction, and it's lovely to see people coming back and asking how you are. You ask them how they are and how things went with what they bought. It's always a bit like a town square, especially when several customers who don't know each other chat, exchanging ideas, recipes, and advice. [P_13]*

These eco-entrepreneurs recognize the environmental and social implications of capitalism, prompting them to rethink the concept of entrepreneurship. They believe that entrepreneurs should prioritize environmental, social, and ethical goals. This reimagining involves discovering new ways to balance present needs with future aspirations and integrating everyday practices with a hopeful vision for the future.

## 5 Discussion

Let us now discuss the key points that emerged from our analysis: motivations for ecopreneurship (becoming); how the business is organized (doing); and the transformative power of everyday practices (making).

Becoming an ecopreneur goes beyond simply assessing economic opportunities or filling a niche in an emerging market. Similarly, it is too simplistic to attribute this choice solely to an “eco-habitus” already embedded in the interviewees' daily practices and value orientations. Instead, the decision to become an ecopreneur should be viewed as a significant biographical turning point: the discrepancy between their beliefs and their livelihood motivates them to critically re-examine the past and redefine their expectations for the future. This process aims to restore a sense of coherence and continuity in their biographies. These discontinuities serve as “catalysts for reflexivity,” encouraging a reassessment of daily practices. The biographical rupture does not replace the eco-habitus; rather, it reveals how it can be translated into an entrepreneurial project. To bridge this biographical discontinuity, ecopreneurs draw upon their capacity for agency in response to both structural constraints, such as unemployment and a lack of welfare, and significant global challenges that politics struggles to address, such as the climate crisis and social justice issues. Specifically, the pursuit of greater authenticity serves as a means of moral responsibility, leading to new forms of individualized solidarity (Berking, 1996). This finding adds to and complicates the concept of “eco-habitus,” confirming that a mindset embedded in critical consumption practices can transform into an entrepreneurial project. However, this comes with the risk of placing excessive responsibility on individuals, forcing them to craft their own life narratives while also trying to reconnect the environment, the economy, and society—three spheres that capitalism tends to keep separate. This underscores the value of our micro-relational perspective.

Doing ecopreneurship relates to the way a business is organized, including its choice of suppliers and its relationships with employees and customers. This allows us to view sustainability not as an abstract principle, but as a contextualized organizational practice that reveals its limitations and contradictions. Ecopreneurs continually redefine economic relationships to detach them from the logic of globalized capitalism. The interviewees describe working environments where relationships are founded on trust, recognition, and reciprocity. They favor local producers and regional (or neighboring) industrial clusters over globalized supply chains, and, when sourcing from abroad, they rely on sustainability certifications. As a result, the choice of suppliers is not based solely on price and product quality. These are often long-standing relationships supported by a shared set of values. In their relationships with employees, ecopreneurs prioritize contractual guarantees, positive colleagues' interactions, and the promotion of a healthy work-life balance. The interviewees portray entrepreneurs who are open to dialogue and discussion, even willing to share decision-making with their employees to some extent. Similarly, in their relationships with customers, we register exchanges and forms of social interaction that transcend purely functional roles. Doing ecopreneurship illustrates that sustainability is never fully realized, but is continually created, assessed, and negotiated within everyday economic practices.

Making ecopreneurship is characterized by the commitment to pursuing economic, environmental, and social objectives simultaneously. Ecopreneurs also demonstrate the ability to imagine and implement innovative prefigurative economic practices. To varying degrees, all those interviewed challenge the current economic system, including globalized supply chains, job insecurity, pricing logic, and the commodification of urban life. It is precisely this capacity for prefiguration that enables them to avoid the risk of “presentification”: they aspire to a different world and prefigure a real utopia that takes shape in everyday practices. They find the urban context to be the ideal environment for experimenting with real utopias in daily life (Wright, 2010; Cooper, 2014). They tap into the latent potential of the present, seeking alternatives that can be realized in the here and now within the fabric of daily life. By doing so, the implement micro-practices that harness NUEs' transformative potential. These concrete actions not only foreshadow the desired future but also capture the temporal dimension of utopia (Wright, 2010; Levitas, 2013; Cooper, 2014). The utopia envisioned by ecopreneurs is not a distant future; it takes shape in everyday activities. The hope for a different future—one that is more environmentally and socially sustainable—is reimagined in both subjective and collective terms. This emerges as innovative and creative practices that challenge conventional norms and open up new possibilities. In this way, ecopreneurs uniquely connect the present with the future, linking their everyday practices with their hopes for a better future.

To conclude, everyday life provides a lens for studying NUE because it reveals ecopreneurs' motivations and the organizational and utopic practices that distinguish these ventures from traditional firms. While sustainable entrepreneurship has been examined through macro-level categories such as innovation, environmental impact, and business models (Schaltegger and Wagner, 2011; Muñoz and Cohen, 2017), its micro-practices remain underexplored. Our first contribution is to address this gap by showing how change is produced and negotiated in everyday organizational life. Second, the article shifts attention from forecasting strategies (Poli, 2024) to everyday life as the site in which real utopian projects are enacted (Wright, 2010). Amid uncertainty and social acceleration (Rosa, 2013), NUE practices connect past experiences, present constraints, and imagined futures, grounding action in responsibility toward future generations (Jonas, 1979). These practices are transformative not because they eliminate contradictions, but because they turn them into objects of experimentation. Ecopreneurs struggle to rely on local supply chains, provide secure employment, and maintain affordable prices. Aware that they confront the constraints of capitalism (P_23), they neither identify with the Schumpeterian destroyer nor embrace the heroic entrepreneur who saves the planet. Their work may be experienced as a mission, but not as evidence of exceptional individual power. The point is not to attribute to ecopreneurs a heroic capacity for systemic transformation, but to show how they make alternative forms of economic exchange practicable. Recycling and reuse workshops, relations with employees, and informal exchanges with customers enact an economy centered on solidarity, social ties, decent work, and environmental responsibility. These situated experiments also expand imaginable “futurescapes” and possible strategies for economic action.

The article's main contribution is thus to locate the transformative potential of NUE beyond macro-level outcomes. It also lies in the capacity of everyday routines to produce, reproduce, and negotiate sustainability, while making alternative ways of reconciling economy, society, and the environment legitimate and practicable.

## Data Availability

The raw data supporting the conclusions of this article will be made available by the authors, without undue reservation.

## References

[B1] Adam B. (1990). Time and Social Theory. Cambridge: Polity Press.

[B2] Adam B. (2003). When time is money: contested rationalities of time in the theory and practice of work. Theoria, 50, 94–125. doi: 10.3167/004058103782267403

[B3] Appadurai A. (2004). “The capacity to aspire: culture and the terms of recognition,” in Culture and Public Action, eds. V. Rao, and M. Walton (Palo Alto, CA: Stanford University Press), 59–84.

[B4] Azzolina L. Mazzenzana S. (2026). Sustainable entrepreneurship between ecological distinction and local context. Front. Sociol. 11:1816778. doi: 10.3389/fsoc.2026.1816778 42487667 PMC13388119

[B5] Bagnasco A. (1977). Tre Italie. La problematica territoriale dello sviluppo italiano. Bologna: Il Mulino.

[B6] Bandelj N. (2020). Relational work in the economy. Annu. Rev. Sociol. 46, 251–272. doi: 10.1146/annurev-soc-121919-054719

[B7] Bansal P. DesJardine M.R. (2014). Business sustainability: it is about time. Strateg. Organ. 12, 70–78. doi: 10.1177/1476127013520265

[B8] Bansal P. Reinecke J. Suddaby R. Langley A. (2022). Temporal work: the strategic organization of time. Strateg. Organ. 20, 6–19. doi: 10.1177/14761270221081332

[B9] Battilani P. Zamagni V. (2012). The managerial transformation of Italian co-operative enterprises 1946–2010. Bus. Hist. 54, 964–985. doi: 10.1080/00076791.2012.706893

[B10] Beckert J. (2016). Imagined Futures: Fictional Expectations and Capitalist Dynamics. Cambridge, MA: Harvard University Press. doi: 10.4159/9780674545878

[B11] Berking H. (1996). “Solidary individualism: the moral impact of cultural modernisation in late modernity,” in Risk, Environment and Modernity: Towards a New Ecology, eds. S. Lash, B. Szerszynski, and B. Wynne. London: SAGE Publications, 189–202. doi: 10.4135/9781446221983.n9

[B12] Block F. Somers M. R. (2014). The Power of Market Fundamentalism: Karl Polanyi's Critique. Cambridge, MA: Harvard University Press. doi: 10.4159/harvard.9780674416345

[B13] Botelho T. L. Gulati R. Sorenson O. (2024). The sociology of entrepreneurship revisited. Annu. Rev. Sociol. 50, 341–364. doi: 10.1146/annurev-soc-030222-014049

[B14] Bourdieu P. (1984) Distinction: A Social Critique of the Judgement of Taste. Cambridge, MA: Harvard University Press..

[B15] Carfagna L.B. Dubois E.A. Fitzmaurice C. Ouimette M.Y. Schor J.B. Willis M. . (2014). An emerging eco-habitus: the reconfiguration of high cultural capital practices among ethical consumers. J. Consum. Cult. 14, 158–178. doi: 10.1177/1469540514526227

[B16] Clarke J. (2007). Unsettled connections. Citizens, consumers and the reform of public services. J. Consum. Cult. 7, 159–178.

[B17] Cohen B. Muñoz P. (2015). Toward a theory of purpose-driven urban entrepreneurship. Organ. Environ. 28, 264–285. doi: 10.1177/1086026615600883

[B18] Cooper D. (2014). Everyday Utopias: The Conceptual Life of Promising Spaces. Durham, NC: Duke University Press. doi: 10.1215/9780822377153

[B19] De Clercq D. Voronov M. (2011). Sustainability in entrepreneurship: a tale of two logics. Int. Small Bus. J. 29, 322–344. doi: 10.1177/0266242610372460

[B20] Duranton G. Puga D. (2001). Nursery cities: urban diversity, process innovation, and the life cycle of products. Am. Econ. Rev. 91, 1454–1477. doi: 10.1257/aer.91.5.1454

[B21] Florida R. Adler P. Mellander C. (2017). The city as innovation machine. Reg. Stud. 51, 86–96. doi: 10.1080/00343404.2016.1255324

[B22] Fontana C. Testi A. Allegretti G. Zetti I. Rossi M. (2025). Embedding justice into climate policies through participatory approaches: prospects and pitfalls toward community-based adaptation pathways in Bologna, Italy. Cities 163:105998. doi: 10.1016/j.cities.2025.105998

[B23] Forno F. Graziano P.R. (2014). Sustainable community movement organizations. J. Consum. Cult. 14, 139–157. doi: 10.1177/1469540514526225

[B24] Gibson-Graham J.K. (2008). Diverse economies: performative practices for ‘other worlds'. Prog. Hum. Geogr. 32, 613–632. doi: 10.1177/0309132508090821

[B25] Ginsburger M. (2020). Eco-citizenship: from norm to practice. Rev. Fr. Sociol. 61, 43–78. doi: 10.3917/rfs.611.0043

[B26] Giovanardi M. Silvagni M.G. (2021). Profiling “Red Bologna”: between neoliberalisation tendencies and municipal socialist legacy. Cities 110:103059. doi: 10.1016/j.cities.2020.103059

[B27] Halberstadt J. Schwab A.K. Kraus S. (2024). Cleaning the window of opportunity: towards a typology of sustainability entrepreneurs. J. Bus. Res. 171:114386. doi: 10.1016/j.jbusres.2023.114386

[B28] Hassard J. (2017). “Time and industrial sociology,” in Time, Work and Organization, eds. J. Hassard, R. Holliday, and H. Willmott (London: Routledge), 13–34. doi: 10.4324/9781315267272-2

[B29] Hayes A.S. (2024). Time, ties, transactions: temporality and relational work in economic exchange. Theory Soc. 53, 625–651. doi: 10.1007/s11186-024-09552-9

[B30] Hirschman D. (2021). Transitional temporality. Sociol. Theory 39, 48–58. doi: 10.1177/0735275120981048

[B31] Honneth A. (2019). Riconoscimento. Storia di un'idea europea. Milano: Feltrinelli.

[B32] Istat (2026). Censimento Popolazione Emilia Romagna. Available online at: https://www.istat.it/wp-content/uploads/2026/04/08_Focus_Censimento_Popolazione_Emilia_Romagna_2024.pdf (Accessed May 14, 2026).

[B33] Jameson F. (2005). Archaeologies of the Future: The Desire Called Utopia and Other Science Fictions. London: Verso.

[B34] Jonas H. (1979). Das Prinzip Verantwortung. Versuch einer Ethik für die technologische Zivilisation. Frankfurt am Main: Insel Verlag.

[B35] Johnston J. (2008) The citizen-consumer hybrid: ideological tensions and the case of whole foods market. Theor. Soc. 37, 229–270. 10.1007/s11186-007-9058-5.

[B36] Kaplan S. Orlikowski W.J. (2013). Temporal work in strategy making. Organ. Sci. 24, 965–995. doi: 10.1287/orsc.1120.0792

[B37] Kennedy E. Givens J. (2019). Eco-habitus or eco-powerlessness? Examining environmental concern across social class. Sociol. Perspect. 62, 646–667. doi: 10.1177/0731121419836966

[B38] Levitas R. (2013). Utopia as a Method: The Imaginary Reconstitution of Society. Basingstoke: Palgrave Macmillan.

[B39] Logan J.R. Molotch H. (1987). Urban Fortunes: The Political Economy of Place. Berkeley and Los Angeles, CA: University of California Press.

[B40] Melo P. C. Graham D. J. Noland R. B. (2009). A meta-analysis of estimates of urban agglomeration economies. Reg. Sci. Urban Econ. 39, 332–342.

[B41] Micheletti M. (2003). Political Virtue and Shopping: Individuals, Consumerism and Collective Action. New York: Palgrave Macmillan. doi: 10.1057/9781403973764

[B42] Miyazaki H. (2003). The temporalities of the market. Am. Anthropol. 105, 255–265. doi: 10.1525/aa.2003.105.2.255

[B43] Monticelli L. (2018). Embodying alternatives to capitalism in the 21st century. tripleC 16, 501–517. doi: 10.31269/triplec.v16i2.1032

[B44] Muñoz P. Cohen B. (2017). Sustainable entrepreneurship research: taking stock and looking ahead. Bus. Strategy Environ. 27, 300–322. doi: 10.1002/bse.2000

[B45] Muñoz P. Dimov D. (2015). The call of the whole in understanding the development of sustainable ventures. J. Bus. Ventur. 30, 632–654. doi: 10.1016/j.jbusvent.2014.07.012

[B46] Polanyi K. (2002). “The great transformation,” in Readings in Economic Sociology, ed. F. Dobbin (Princeton, NJ: Princeton University Press), 38–62. doi: 10.1002/9780470755679.ch4

[B47] Poli R. (ed.). (2024). Handbook of Futures Studies. Cheltenham: Edward Elgar Publishing.

[B48] Rindova V.P. Martins L.L. (2022). Futurescapes: imagination and temporal reorganization in the design of strategic narratives. Strateg. Organ. 20, 200–224. doi: 10.1177/1476127021989787

[B49] Rosa H. (2013). Social Acceleration: A New Theory of Modernity. New York, NY: Columbia University Press. doi: 10.7312/rosa14834

[B50] Sánchez-Hernández J.L. Glückler J. (2019). Alternative economic practices in Spanish cities: from grassroots movements to urban policies? An institutional perspective. Eur. Plan. Stud. 27, 2450–2469. doi: 10.1080/09654313.2019.1644295

[B51] Sartori L. (2020). The social life of Sardex and Liberex: kin or acquaintances? A comparison between two mutual credit circuits in Italy. Partecip. Conflitto 2, 487–513. doi: 10.1285/i20356609v13i1p487

[B52] Sassatelli R. (2006). “Virtue, responsibility and consumer choice,” in Consuming Cultures, Global Perspectives: Historical Trajectories, Transnational Exchanges, eds. J. Brewer, and F. Trentmann (Oxford: Berg), 219–250.

[B53] Schaltegger S. Wagner M. (2011). Sustainable entrepreneurship and sustainability innovation: categories and interactions. Bus. Strategy Environ. 20, 222–237. doi: 10.1002/bse.682

[B54] Schlange L.E. (2009). Stakeholder identification in sustainability entrepreneurship. Greener Manag. Int. 55, 13–32. doi: 10.9774/GLEAF.3062.2006.au.00004

[B55] Schütz A. Luckmann T. (1974). The Structure of the Life World. Evanston, IL: Northwestern University Press.

[B56] Shepherd D.A. Patzelt H. (2011). The new field of sustainable entrepreneurship: studying entrepreneurial action linking “what is to be sustained” with “what is to be developed”. Entrep. Theory Pract. 35, 137–163. doi: 10.1111/j.1540-6520.2010.00426.x

[B57] Shove E. (2020). “Everyday practice and the production and consumption of time,” in Time, Consumption and Everyday Life, eds. E. Shove, F. Trentmann, and R. Wilk (London: Routledge), 17–33. doi: 10.4324/9781003087236-3

[B58] Shrivastava P. Kennelly J.J. (2013). Sustainability and place-based enterprise. Organ. Environ. 26, 83–101. doi: 10.1177/1086026612475068

[B59] Vale M. Peponi A. Carvalho L. Veloso A.P. Queirós M. Morgado P. (2024). Are peripheral regions in troubled waters for sustainability transitions? A systematic analysis of the literature. Eur. Urban Reg. Stud. 31, 116–131. doi: 10.1177/09697764231194316

[B60] World Commission on Environment and Development (WCED) (1987). Our Common Future. New York, NY: Oxford University Press.

[B61] Wright E.O. (2010). Envisioning Real Utopias. London: Verso.

[B62] York J.G. O'Neil I. Sarasvathy S.D. (2016). Exploring environmental entrepreneurship: identity coupling, venture goals, and stakeholder incentives. J. Manag. Stud. 53, 695–737. doi: 10.1111/joms.12198

[B63] Young C. Tilley F. (2006). Can businesses move beyond efficiency? The shift towards effectiveness and equity in the corporate sustainability debate. Bus. Strategy Environ. 15, 402–415. doi: 10.1002/bse.510

[B64] Zanoni P. Contu A. Healy S. (2017). Post-capitalistic politics in the making: the imaginary and praxis of alternative economies. Organization 24, 575–588. doi: 10.1177/1350508417713219

[B65] Zelizer V.A. (2004). “Circuits of commerce,” in Self, Social Structure, and Beliefs: Explorations in Sociology, eds. J. C. Alexander, G. T. Marx, and C. L. Williams (Berkeley, CA: University of California Press), 122–144. doi: 10.1525/california/9780520241367.003.0009

[B66] Zelizer V.A. (2012). How I became a relational economic sociologist and what does that mean?. Polit. Soc. 40, 145–174. doi: 10.1177/0032329212441591

[B67] Zerubavel E. (1985). Hidden Rhythms: Schedules and Calendars in Social Life. Berkeley, CA: University of California Press.

